# Social patterning of overeating, binge eating, compensatory behaviours and symptoms of bulimia nervosa in young adult women: results from the Australian Longitudinal Study on Women’s Health

**DOI:** 10.1017/S1368980016001440

**Published:** 2016-06-22

**Authors:** Ilona Koupil, Leigh Tooth, Amy Heshmati, Gita Mishra

**Affiliations:** 1 Centre for Health Equity Studies (CHESS), Stockholm University/Karolinska Institutet, Sveavagen 160, SE 10691 Stockholm, Sweden; 2 Department of Public Health Sciences, Karolinska Institutet, Stockholm, Sweden; 3 School of Public Health, Faculty of Medicine and Biomedical Sciences, The University of Queensland, Herston, Queensland, Australia

**Keywords:** Disordered eating, Binge eating, Bulimia nervosa, Overeating, Social determinants

## Abstract

**Objective:**

To study social patterning of overeating and symptoms of disordered eating in a general population.

**Design:**

A representative, population-based cohort study.

**Setting:**

The Australian Longitudinal Study on Women’s Health (ALSWH), Survey 1 in 1996 and Survey 2 in 2000.

**Subjects:**

Women (*n* 12 599) aged 18–23 years completed a questionnaire survey at baseline, of whom 6866 could be studied prospectively.

**Results:**

Seventeen per cent of women reported episodes of overeating, 16 % reported binge eating and 10 % reported compensatory behaviours. Almost 4 % of women reported symptoms consistent with bulimia nervosa. Low education, not living with family, perceived financial difficulty (OR=1·8 and 1·3 for women with severe and some financial difficulty, respectively, compared with none) and European language other than English spoken at home (OR=1·5 for European compared with Australian/English) were associated with higher prevalence of binge eating. Furthermore, longitudinal analyses indicated increased risk of persistent binge eating among women with a history of being overweight in childhood, those residing in metropolitan Australia, women with higher BMI, smokers and binge drinkers.

**Conclusions:**

Overeating, binge eating and symptoms of bulimia nervosa are common among young Australian women and cluster with binge drinking. Perceived financial stress appears to increase the risk of binge eating and bulimia nervosa. It is unclear whether women of European origin and those with a history of childhood overweight carry higher risk of binge eating because of genetic or cultural reasons.

Eating disorders (ED), anorexia nervosa (AN), bulimia nervosa (BN) and related disorders are one of the leading causes of disease burden in terms of years of life lost through death or disability in young women^(^ ^1^ ^)^. Although they are relatively uncommon, ED have severe consequences for patients, including increased mortality^(^ ^2^ ^)^, and are associated with significant psychiatric and physical complications, including high risk of pregnancy complications^(^ ^3^ ^,^ ^4^ ^)^.

Studies of social patterning of ED tend to focus on AN alone^(^ ^5^ ^,^ ^6^ ^)^ or upon ED as a group^(^ ^7^ ^)^, with less research on BN or milder symptoms of disordered eating. Having reviewed research published in the 1970s to the early 1990s, Gard and Freeman^(^ ^8^ ^)^ concluded the relationship between AN and high socio-economic status still had to be proved, while there was increasing evidence to suggest a negative association between socio-economic group and risk of BN. Similarly, in her review of social patterning of ED, Gibbons^(^ ^9^ ^)^ emphasised that ED are not limited to upper social classes and concluded that women from lower socio-economic groups exhibit more signs of disordered eating behaviour.

An earlier study based on data from the Australian Longitudinal Study on Women’s Health (ALSWH) showed generally lower prevalence of symptoms of ED in immigrant women and a strong acculturation effect in risk of ED becoming more similar to the majority population with increasing length of stay^(^ ^10^ ^)^. Those findings are consistent with results from other countries: Sinha and Warfa^(^ ^11^ ^)^ reviewed patterns of health-care seeking and consumption among ethnic minority communities in the UK and USA, and showed that women from ethnic minorities were far less likely to seek and receive treatment for their ED and also less likely to be diagnosed and referred to ED services or clinics for treatment of their ED.

Information about social patterning of signs of disordered eating behaviour and untreated ED is helpful in identifying groups at risk and those in need of intervention. Hay *et al*.^(^ ^12^ ^,^ ^13^ ^)^ studied the prevalence of binge eating, purging and strict dieting or fasting for weight or shape control among the general population of South Australia and reported a twofold increase in the prevalence of these disordered eating behaviours between 1995 and 2005. The authors also documented strong age and gender differences but did not explore variation in prevalence of behaviours across socio-economic groups in Australia^(^ ^13^ ^)^.

We investigated the burden and social patterning of overeating and symptoms of disordered eating among women from a large population based study, the ALSWH, who were born between 1973 and 1978 and whose symptoms and behaviours were measured in 1996 (age 18–23 years) and 2000 (age 22–27 years). We also studied associations of sociodemographic, reproductive and behavioural characteristics with persistence of disordered eating symptoms during young adulthood.

## Methods

The ALSWH^(^ ^14^ ^)^ is a national longitudinal study of factors affecting the health and well-being of four cohorts of women who were born in 1921–26, 1946–51, 1973–78 and 1989–95. The study provides longitudinal data on health and well-being, health-service use, sociodemographic characteristics and health behaviour collected by self-completed questionnaires.

The original ALSWH sample (1921–26, 1946–51, 1973–78 cohorts) was selected randomly from the Medicare Health Insurance Commission database (which includes all residents of Australia regardless of age, including immigrants and refugees), with intentional over-representation of women from rural and remote areas. Informed consent was obtained from all participants at each survey, with ethical clearance obtained from the University of Newcastle and the University of Queensland in Australia. Further details of the recruitment methods are described elsewhere^(^ ^15^ ^,^ ^16^ ^)^. Since the baseline survey in 1996, the three age cohorts have been surveyed annually on a rolling basis. The 1989–95 cohort was recruited in 2012/13 and is surveyed annually.

The present study used data from the 1973–78 cohort. A total of 14 247 women (estimated 42 % response rate) participated in Survey 1 in 1996, of whom 9604 (69 %) also completed Survey 2 in 2000. Comparisons with the 1999 and 2001 Australian Censuses reveal that while there is some over-representation among these cohorts of women with a higher educational qualification, the study remains broadly representative of women in the general population^(^ ^17^ ^)^.

### Measures

#### Outcome

Six outcome variables were derived using a series of questions originally designed to reflect the diagnostic criteria for ED disorders established by the American Psychiatric Association and presented in the *Diagnostic and Statistical Manual of Mental Disorders, Fourth Edition (DSM-IV)* ^(^ ^18^ ^)^. At Survey 1 the women were asked the following questions: ‘Have there been times when you felt that you have eaten what other people would regard as an unusually large amount of food given the circumstances?’; ‘During these times of overeating did you have a sense of having lost control over your eating, that is, feeling that you couldn’t stop eating once you had started?’; and ‘Have you used any of the following to control your weight or shape?’ (i) vomited on purpose after eating, (ii) laxatives, (iii) diuretics, (iv) fasting (not eating food for at least a day). The response options for all above questions included: ‘yes, in the past month’, ‘yes, more than one month ago’ and ‘no’, and we used the information about symptoms during the past month to generate variables indicating prevalent disordered eating.

The derived outcome variables at Survey 1 were: (i) overeating episodes (without lost control of eating behaviour); (ii) binge eating (episodes of overeating with lost control); (iii) compensatory weight-control behaviours (purposeful vomiting, use of laxatives, use of diuretics or fasting for at least one day); and (iv) symptoms of BN (binge eating with compensatory behaviours such as purposeful vomiting, use of laxatives, use of diuretics or fasting for at least one day).

Two additional outcome variables were derived to capture ‘transitions’ in overeating and binge eating using the questions that were asked identically at Surveys 1 and 2. These were: (v) overeating episodes recorded as persistent across Surveys 1 and 2, transient (ever at Survey 1 or 2) or never; and (vi) binge eating (episodes of overeating with lost control) recorded as persistent across Surveys 1 and 2, transient (ever at Survey 1 or 2) or never. It was not possible to derive transitions in compensatory weight-control behaviours or symptoms of BN as the response options at the two surveys were not identical. Women who were pregnant at the time of the survey were not included in these analyses.

#### Exposures

Own highest education (≤12 years or equivalent (e.g. higher school certificate); trade/apprenticeship/certificate/diploma; university/higher university degree), employment status, ability to manage on available income (ranked on 5-point scale ranging from ‘It is impossible’ to ‘It is easy’), area of residence (classified according to the Australian Standard Geographic Classification for remoteness based on road distance to the closest service centre, which in turn is categorised on population size as: major cities of Australia; inner regional Australia; outer regional Australia; remote/very remote Australia)^(^ ^19^ ^)^, living situation (partner/children; alone; parents/others; non-family), parity (no children; one child; two or more children), language spoken at home (derived variable based on responses to questions on country of birth and language: English Australian; English other; European; Asian/other) and age at Survey 1 (18–23 years). These variables were measured at Survey 1.

Highest educational qualification attained by their mother/step-mother and father/step-father (or other main caregivers while they were growing up) was measured in Survey 2. We combined maternal and paternal education into a variable indicating highest education of the parent/s and recoded this into four categories: ≤12 years; trade/apprenticeship/certificate/diploma; university/higher university degree; and not known. Self-reported height and weight were used to compute BMI (= [weight (kg)]/[height (m)]^2^). BMI was categorised as: underweight (BMI<18·5 kg/m^2^); normal weight (BMI=18·5–24·9 kg/m^2^); and overweight/obese (BMI≥25·0 kg/m^2^)^(^ ^20^ ^)^. Cigarette smoking status was defined as: never smoked; ex-smoker; or current smoker. Binge alcohol consumption was originally coded as ‘never’, ‘less than once a month’, ‘about one a month’ and ‘at least once a week’ and recoded into at least once per week *v*. less often. Self-reported weight as a child was classified as underweight (included very underweight and slightly underweight), average or overweight (included slightly overweight and very overweight). These variables were measured at Survey 1.

### Missing data

Data on sociodemographic characteristics were almost complete; however, there was a larger proportion of missing data on BMI (14 %) and smoking (4 %). Information on disordered eating was relatively complete and we could generate the respective outcome measures for 98 % of the sample. We further excluded 419 women who reported being pregnant at Survey 1 and 484 women at Survey 2.

Complete data on all variables used in analyses of Survey 1 were available for 12 599 non-pregnant women (88 % of all respondents). Data for analyses on health and behavioural characteristics in relation to persistent overeating and persistent binge eating were available for 6866 women who were not pregnant in either survey (71 % of Survey 2 respondents). Comparisons of sociodemographic characteristics and prevalence of symptoms and behaviours between the samples of complete cases and those excluded from analyses indicated that women with higher education and those without financial difficulties were over-represented in our analytical sample, and the overall prevalence of overeating and binge eating in analyses of data from the first survey was underestimated by up to 1 %.

### Statistical analyses

Chi-square tests were used for descriptive analyses. First, the prevalence of the four main outcomes (overeating, binge eating, compensatory behaviours and symptoms consistent with BN) were determined and then their associations with sociodemographic characteristics were analysed by *χ* ^2^ and logistic regression. Each outcome was analysed separately in logistic regression adjusted for age. Characteristics significantly associated with each of the outcomes were entered into multivariable models. Univariable and multivariable multinominal regressions were used to study associations with persistent and transient overeating and binge eating, respectively. The first set of analyses included sociodemographic characteristics measured in early adulthood (at Survey 1) and outcomes generated from questions asked at Survey 1. Subsequent analyses further explored associations of childhood exposures (parental education and weight in childhood) in relation to persistence of symptoms between Surveys 1 and 2. Statistical analyses were conducted using the statistical software packages SAS version 9.3 and STATA version 11.

## Results

Table 1 presents the distributions of sociodemographic characteristics of the women at Survey 1; the mean age of women was 20·8 (sd 1·5) years, just over a half lived in major cities, 92 % reported speaking English at home, 70 % had up to 12 years of education and about half of the women were in paid work. Approximately 50 % of women lived with parents or relatives and only 10 % had children. The overall prevalence of overeating or binge eating was 33·0 %, with 17·4 % of the women reporting overeating episodes (i.e. overeating without a sense of having lost control over their eating) and 15·6 % of the women reporting binge eating. Compensatory measures for weight control were reported by 9·8 % of the women and 3·6 % of women reported symptoms consistent with BN.

**Table 1:** Prevalence of overeating, binge eating, extreme weight-control behaviours and symptoms consistent with bulimia nervosa, by sociodemographic characteristics, among 12 599 Australian women aged 18–23 years, Australian Longitudinal Study on Women’s Health

			Overeating	Binge eating	Compensatory behaviours	Bulimia nervosa*
	*n*	%	%	%	%	%
Residence						
Major cities	6589	52·3	17·0	**16**·**7**	10·0	**4**·**1**
Inner regional	3808	30·2	18·1	**13**·**9**	9·8	**3**·**3**
Outer regional	1824	14·5	17·0	**14**·**6**	9·0	**2**·**7**
Remote	378	3·0	17·7	**15**·**9**	8·5	**2**·**4**
*P* value			0·545	**0**·**001**	0·475	**0**·**011**
Language						
English, Australian	10 948	86·9	17·6	**15**·**3**	**9**·**5**	3·5
English, other	647	5·1	17·0	**16**·**1**	**8**·**5**	3·1
European	520	4·1	16·1	**21**·**1**	**15**·**2**	6·1
Asian or other	484	3·8	14·9	**14**·**7**	**11**·**4**	3·3
*P* value			0·398	**0**·**004**	**0**·**001**	0·015
Education						
Up to 12 years	8842	70·2	17·3	**16**·**4**	**10**·**4**	3·8
Trade/certificate/diploma	2302	18·3	18·1	**14**·**0**	**9**·**7**	3·3
Degree or higher degree	1455	3·8	16·7	**12**·**9**	**5**·**8**	3·0
*P* value for linear trend			0·527†	**0**·**001**	**0**·**001**	0·216
Employment						
Paid work	6641	52·7	**18**·**5**	15·5	**9**·**5**	3·5
Unemployed	867	6·9	**16**·**1**	16·7	**16**·**0**	4·8
Other	5091	40·4	**16**·**1**	15·5	**9**·**1**	3·5
*P* value			**0**·**002**	0·617	**0**·**001**	0·131
Financial difficulty						
Not too bad/easy	6149	48·8	**16**·**3**	**12**·**8**	**7**·**2**	**2**·**6**
Difficult sometimes	4164	33·0	**18**·**0**	**16**·**6**	**10**·**5**	**3**·**7**
Difficult always	2286	18·1	**18**·**9**	**21**·**0**	**15**·**2**	**6**·**1**
*P* value for linear trend			**0**·**007**	**0**·**001**	**0**·**001**	**0**·**001**
Living arrangements						
Parents/relatives	6221	49·4	**16**·**9**	**15**·**0**	**9**·**2**	3·5
Partner and/or children	3401	27·0	**16**·**8**	**15**·**1**	**10**·**0**	3·3
Non-family	2197	17·4	**18**·**7**	**17**·**6**	**10**·**0**	4·1
Alone	780	6·2	**20**·**0**	**16**·**5**	**12**·**6**	4·7
*P* value			**0**·**039**	**0**·**023**	**0**·**024**	0·133
Parity						
No children	11 454	90·9	17·4	15·5	**9**·**5**	3·6
One child	829	6·6	17·0	16·6	**12**·**5**	4·1
Two or more children	316	2·5	15·2	14·6	**11**·**1**	3·5
*P* value			0·555	0·603	**0**·**013**	0·733
Age (years)						
18	448	3·6	19·0	15·8	**12**·**7**	4·2
19	2691	21·4	17·3	16·5	**10**·**7**	3·9
20	2500	19·8	18·8	15·9	**10**·**1**	3·2
21	2484	19·7	17·4	15·7	**9**·**6**	3·7
22	2467	19·6	16·8	14·6	**9**·**2**	3·8
23	2009	16·0	15·9	14·8	**8**·**4**	3·1
*P* value for linear trend			0·156	0·053	**0**·**001**	0·329
TOTAL	12 599	100·0	17·4	15·6	9·8	3·6
			(*n* 2188)	(*n* 1960)	(*n* 1230)	(*n* 455)

Significant results are indicated in bold font.

* Symptoms consistent with bulimia nervosa.

† *P* value for heterogeneity.

### Cross-sectional analyses

In univariable analyses (Table 1), self-reported financial difficulty was most strongly associated with higher prevalence of all the studied outcomes. Lower education was also strongly associated with higher prevalence of binge eating and compensatory behaviours. Furthermore, we noted a higher prevalence of binge eating, compensatory behaviours and symptoms consistent with BN in women from homes where a European language other than English was spoken; a higher prevalence of binge eating and BN symptoms among women living in major cities; and a higher prevalence of compensatory behaviours among women with children and those who were unemployed. Living alone or with others than family members was also associated with higher risk of overeating, binge eating and compensatory behaviours. We observed a strong negative association between age and the prevalence of compensatory behaviours.

Cross-sectional multivariable analyses using data from Survey 1 confirmed a strong association of perceived financial difficulty with higher prevalence of all studied outcomes; there was a particularly high risk of compensatory behaviours and symptoms consistent with BN among those with the highest degree of financial stress (Table 2). Additionally, binge eating was more common in women with lower education and those not living with their family. The prevalence of overeating episodes appeared to be lower among women without paid work and the prevalence of compensatory behaviours was highest among the unemployed. Women who did not live with their family also had higher risk of compensatory behaviours, while living in regional Australia was associated with slightly lower risk of binge eating and symptoms of BN. Lastly, European-language speakers consistently showed a substantially higher risk of binge eating, compensatory behaviours and symptoms consistent with BN in all age-adjusted and multivariable analyses (Table 2).

**Table 2:** Odds ratios for overeating, binge eating, extreme weight-control behaviours and symptoms consistent with bulimia nervosa, by sociodemographic characteristics, among 12 599 Australian women aged 18–23 years, Australian Longitudinal Study on Women’s Health. Age-adjusted and multivariable analyses

	Overeating (*n* 2188)				Binge eating (*n* 1960)				Compensatory behaviours (*n* 1230)				Bulimia nervosa* (*n* 455)			
	Model 1		Model 2		Model 1		Model 2		Model 1		Model 2		Model 1		Model 2	
	OR	95 % CI	OR	95 % CI	OR	95 % CI	OR	95 % CI	OR	95 % CI	OR	95 % CI	OR	95 % CI	OR	95 % CI
Residence																
Major cities	1		–		**1**		**1**		1		–		**1**		**1**	
Inner regional	1·07	0·97, 1·19			**0**·**80**	**0**·**72**, **0**·**90**	**0**·**78**	**0**·**69**, **0**·**88**	0·97	0·85, 1·11			**0**·**80**	**0**·**64**, **0**·**99**	**0**·**78**	**0**·**63**, **0**·**98**
Outer regional	1·00	0·87, 1·14			**0**·**85**	**0**·**73**, **0**·**98**	**0**·**84**	**0**·**72**, **0**·**98**	0·88	0·74, 1·06			**0**·**66**	**0**·**48**, **0**·**90**	**0**·**67**	**0**·**49**, **0**·**91**
Remote	1·06	0·81, 1·39			**0**·**95**	**0**·**71**, **1**·**26**	**0**·**96**	**0**·**72**, **1**·**28**	0·84	0·58, 1·22			**0**·**57**	**0**·**29**, **1**·**13**	**0**·**59**	**0**·**30**, **1**·**17**
*P* value	0·567				**0**·**001**		**<0**·**001**		0·492				**0**·**009**		**0**·**014**	
Language																
English, Australian	1		–		**1**		**1**		**1**		**1**		**1**		**1**	
English, other	0·97	0·78, 1·19			**1**·**07**	**0**·**86**, **1**·**32**	**1**·**02**	**0**·**82**, **1**·**27**	**0**·**89**	**0**·**67**, **1**·**19**	**0**·**92**	**0**·**69**, **1**·**22**	**0**·**87**	**0**·**55**, **1**·**38**	**0**·**83**	**0**·**52**, **1**·**31**
European	0·91	0·71, 1·15			**1**·**49**	**1**·**20**, **1**·**85**	**1**·**48**	**1**·**18**, **1**·**85**	**1**·**71**	**1**·**34**, **2**·**19**	**1**·**87**	**1**·**45**, **2**·**41**	**1**·**79**	**1**·**24**, **2**·**60**	**1**·**69**	**1**·**16**, **2**·**47**
Asian or other	0·82	0·63, 1·06			**0**·**95**	**0**·**73**, **1**·**23**	**0**·**92**	**0**·**71**, **1**·**20**	**1**·**22**	**0**·**91**, **1**·**62**	**1**·**41**	**1**·**05**, **1**·**89**	**0**·**93**	**0**·**56**, **1**·**55**	**0**·**87**	**0**·**52**, **1**·**45**
*P* value	0·385				**0**·**006**		**0**·**008**		**<0**·**001**		**<0**·**001**		**0**·**031**		**0**·**045**	
Education																
Up to 12 years	1				**1**		**1**		**1**		**1**		1		–	
Trade/certificate/diploma	1·08	0·96, 1·22			**0**·**84**	**0**·**73**, **0**·**96**	**0**·**84**	**0**·**74**, **0**·**97**	**0**·**94**	**0**·**81**, **1**·**10**	**0**·**90**	**0**·**76**, **1**·**05**	0·89	0·68, 1·14		
Degree or higher degree	1·02	0·87, 1·19			**0**·**77**	**0**·**65**, **0**·**91**	**0**·**78**	**0**·**65**, **0**·**93**	**0**·**56**	**0**·**44**, **0**·**72**	**0**·**61**	**0**·**48**, **0**·**79**	0·79	0·56, 1·11		
*P* value for linear trend	0·456†				**<0**·**001**		**0**·**001**		**<0**·**001**		**<0**·**001**		0·130			
Employment																
Paid work	**1**		**1**		1		–		**1**		**1**		1		–	
Unemployed	**0**·**83**	**0**·**68**, **1**·**00**	**0**·**76**	**0**·**62**, **0**·**93**	1·08	0·89, 1·30			**1**·**76**	**1**·**44**, **2**·**14**	**1**·**29**	**1**·**05**, **1**·**59**	1·37	0·98, 1·92		
Other	**0**·**82**	**0**·**75**, **0**·**91**	**0**·**78**	**0**·**71**, **0**·**87**	0·98	0·88, 1·08			**0**·**91**	**0**·**80**, **1**·**03**	**0**·**73**	**0**·**64**, **0**·**84**	0·98	0·80, 1·20		
*P* value	**<0**·**001**		**<0**·**001**		0·609				**<0**·**001**		**<0**·**001**		0·165			
Financial difficulty																
Not too bad/easy	**1**		**1**		**1**		**1**		**1**		**1**		**1**		**1**	
Difficult sometimes	**1**·**12**	**1**·**01**, **1**·**25**	**1**·**16**	**1**·**04**, **1**·**29**	**1**·**34**	**1**·**20**, **1**·**50**	**1**·**34**	**1**·**19**, **1**·**49**	**1**·**49**	**1**·**30**, **1**·**72**	**1**·**46**	**1**·**27**, **1**·**68**	**1**·**43**	**1**·**15**, **1**·**80**	**1**·**46**	**1**·**16**, **1**·**82**
Difficult always	**1**·**19**	**1**·**05**, **1**·**35**	**1**·**26**	**1**·**11**, **1**·**44**	**1**·**80**	**1**·**59**, **2**·**04**	**1**·**77**	**1**·**56**, **2**·**02**	**2**·**26**	**1**·**94**, **2**·**63**	**2**·**15**	**1**·**84**, **2**·**52**	**2**·**40**	**1**·**90**, **3**·**03**	**2**·**42**	**1**·**92**, **3**·**06**
*P* value for linear trend	**0·003**		**<0·001**		**<0·001**		**<0·001**		**<0·001**		**<0·001**		**<0·001**		**<0·001**	
Living arrangements																
Parents/relatives	**1**		**1**		**1**		**1**		**1**		**1**		1		–	
Partner and/or children	**1**·**02**	**0**·**91**, **1**·**14**	**1**·**00**	**0**·**89 1**·**12**	**1**·**04**	**0**·**92**, **1**·**17**	**1**·**01**	**0**·**89**, **1**·**14**	**1**·**18**	**1**·**02**, **1**·**37**	**0**·**99**	**0**·**84**, **1**·**17**	0·99	0·78, 1·25		
Non-family	**1**·**14**	**1**·**00**, **1**·**29**	**1**·**14**	**1**·**01**, **1**·**30**	**1**·**22**	**1**·**07**, **1**·**39**	**1**·**21**	**1**·**06**, **1**·**38**	**1**·**11**	**0**·**94**, **1**·**31**	**1**·**15**	**0**·**97**, **1**·**36**	1·20	0·93, 1·54		
Alone	**1**·**25**	**1**·**04**, **1**·**51**	**1**·**23**	**1**·**02**, **1**·**49**	**1**·**14**	**0**·**93**, **1**·**40**	**1**·**14**	**0**·**93**, **1**·**40**	**1**·**48**	**1**·**18**, **1**·**86**	**1**·**41**	**1**·**12**, **1**·**78**	1·41	0·99, 2·02		
*P* value	**0·039**		**0·037**		**0·024**		**0·027**		**0·005**		**0·017**		0·154			
Parity																
No children	1		–		1		–		**1**		**1**		1		–	
One child	0·99	0·82, 1·19			1·11	0·92, 1·35			**1**·**44**	**1**·**16**, **1**·**78**	**1**·**42**	**1**·**10**, **1**·**83**	1·18	0·82, 1·69		
Two or more children	0·88	0·64, 1·20			0·96	0·70, 1·33			**1**·**30**	**0**·**91**, **1**·**86**	**1**·**35**	**0**·**92**, **1**·**98**	1·01	0·55, 1·87		
*P* value	0·703				0·535				**0·003**		**0·016**		0·681			

Model 1: adjusted for age. Model 2: adjusted for age and mutually adjusted for other variables shown in the column. Significant results are indicated in bold font.

* Symptoms consistent with bulimia nervosa.

† *P* value for heterogeneity.

### Childhood exposures and longitudinal follow-up

Among the 6866 women who were followed longitudinally, 14·8 % reported overeating or binge eating at both surveys (4·7 % overeating at both surveys and 5·8 % binge eating at both surveys) and an additional 31·5 % reported overeating or binge eating at either the first or the second survey (22·8 % overeating and 17·4 % binge eating; Table 3). Univariable analyses indicated stronger social patterning in prevalence of binge eating compared with overeating. Binge drinking and financial difficulty were consistently associated with higher prevalence of both overeating and binge eating. Weight in childhood and at age 18–23 years showed qualitatively different associations with the two outcomes under study in that underweight was associated with a higher prevalence of overeating but a lower prevalence of binge eating, while overweight or obesity was associated with a lower prevalence of overeating and a higher prevalence of binge eating (Table 3).

**Table 3:** Prevalence of transient and persistent overeating episodes and binge eating, by sociodemographic and behavioural characteristics, among 6866 Australian women followed longitudinally from 18–23 years of age, Australian Longitudinal Study on Women’s Health

			Overeating				Binge eating			
	*n*	%	Persistent (%)	Transient (%)	Never (%)	*P* value	Persistent (%)	Transient (%)	Never (%)	*P* value
Parental education										
Up to 12 years	2122	30·9	4·5	23·2	72·2	0·742	**6**·**2**	**17**·**5**	**76**·**3**	**<0**·**001**
Trade/certificate/diploma	1841	26·8	4·8	22·9	72·3		**4**·**4**	**15**·**5**	**80**·**1**	
Degree/higher degree	1686	24·6	5·0	23·4	71·6		**5**·**8**	**17**·**0**	**77**·**2**	
Not known	1217	17·7	4·4	21·1	74·4		**7**·**2**	**20**·**5**	**72**·**2**	
Weight in childhood										
Underweight	1679	24·4	**6**·**2**	**25**·**7**	**68**·**1**	**<0**·**001**	**4**·**0**	**15**·**3**	**80**·**7**	**<0**·**001**
Average	3603	52·5	**4**·**5**	**22**·**4**	**73**·**1**		**4**·**7**	**15**·**7**	**79**·**6**	
Overweight	1584	23·1	**3**·**5**	**20**·**7**	**75**·**8**		**10**·**3**	**23**·**2**	**66**·**4**	
Residence										
Major cities	3664	53·4	4·9	22·0	73·1	0·190	6·5	17·7	75·8	0·059
Inner regional	2043	29·8	5·0	23·3	71·6		5·1	16·5	78·4	
Outer regional	958	13·9	3·3	24·9	71·7		4·3	18·3	77·4	
Remote	201	2·9	4·0	21·9	74·1		7·5	16·4	76·1	
Language										
English, Australian	6012	87·6	4·7	22·8	72·5	0·305	**5**·**7**	**16**·**9**	**77**·**4**	**0**·**005**
English, other	353	5·1	5·9	19·5	74·5		**7**·**4**	**18**·**7**	**73**·**9**	
European	299	4·3	3·0	23·7	73·2		**8**·**0**	**24**·**4**	**67**·**6**	
Asian or other	202	2·9	5·4	26·7	67·8		**4**·**5**	**18**·**3**	**77**·**2**	
Education										
Up to 12 years	4770	69·5	4·6	22·8	72·5	0·978	**6**·**3**	**18**·**2**	**75**·**5**	**0**·**001**
Trade/certificate/diploma	1151	16·8	4·9	23·0	72·1		**4**·**4**	**16**·**4**	**79**·**2**	
Degree or higher degree	945	13·8	5·0	22·3	72·7		**5**·**2**	**14**·**1**	**80**·**7**	
Employment										
Paid work	3578	52·1	4·8	23·2	72·0	0·860	5·7	17·9	76·4	0·723
Unemployed	372	5·4	4·0	21·5	74·5		6·5	18·0	75·5	
Other	2916	42·5	4·7	22·5	72·8		5·9	16·7	77·5	
Financial difficulty										
Not too bad/easy	3604	52·5	**4**·**2**	**21**·**8**	**74**·**0**	**0**·**009**	**4**·**9**	**14**·**9**	**80**·**2**	**<0**·**001**
Difficult sometimes	2167	31·6	**4**·**8**	**23**·**3**	**71**·**9**		**6**·**6**	**19**·**1**	**74**·**3**	
Difficult always	1095	15·9	**6**·**1**	**24**·**9**	**69**·**0**		**7**·**3**	**22**·**2**	**70**·**5**	
Living arrangements										
Parents/relatives	3615	52·6	4·5	22·2	73·4	0·134	5·6	17·3	77·2	0·509
Partner and/or children	1634	23·8	4·1	22·9	73·0		5·4	18·1	76·5	
Non-family	1206	17·6	6·0	24·0	70·0		7·1	17·0	75·9	
Alone	411	6·0	5·4	24·3	70·3		6·1	16·3	77·6	
Parity										
No children	6448	93·9	4·7	22·7	72·6	0·738	5·8	17·3	76·9	0·176
One child	301	4·4	5·0	25·6	69·4		6·0	20·6	73·4	
Two or more children	117	1·7	3·4	22·2	74·4		4·3	11·1	84·6	
Age (years)										
18	243	3·5	7·4	23·5	69·1	0·289	4·9	20·6	74·5	0·313
19	1430	20·8	4·3	23·6	72·1		5·9	18·6	75·5	
20	1344	19·6	5·4	23·5	71·1		5·9	18·8	75·3	
21	1385	20·2	4·8	21·2	74·1		6·3	16·0	77·7	
22	1356	19·8	4·2	22·0	73·8		5·1	16·5	78·4	
23	1108	16·1	4·3	23·8	71·8		6·0	16·1	78·0	
BMI										
Underweight	630	9·2	**5**·**6**	**23**·**2**	**71**·**3**	**0**·**049**	**2**·**9**	**11**·**1**	**86**·**0**	**<0**·**001**
Normal	4738	69·0	**5**·**0**	**23**·**2**	**71**·**8**		**4**·**6**	**16**·**4**	**78**·**9**	
Overweight/obese	1498	21·8	**3**·**5**	**21**·**4**	**75**·**1**		**10**·**7**	**23**·**0**	**66**·**3**	
Smoking										
Non smoker	3986	58·1	4·2	22·2	73·5	0·095	**4**·**7**	**15**·**3**	**80**·**0**	**<0**·**001**
Ex-smoker	970	14·1	5·7	22·7	71·6		**7**·**5**	**19**·**6**	**72**·**9**	
Smoker	1910	27·8	5·2	24·0	70·8		**7**·**3**	**20**·**5**	**72**·**2**	
Alcohol binge drinking										
No	5678	82·7	**4**·**5**	**22**·**4**	**73**·**1**	**0**·**019**	**5**·**3**	**16**·**7**	**78**·**1**	**<0**·**001**
Yes	1188	17·3	**5**·**8**	**24**·**8**	**69**·**4**		**8**·**4**	**20**·**6**	**71**·**0**	
TOTAL	6866	100·0	4·7	22·8	72·5		5·8	17·4	76·8	
			(*n* 323)	(*n* 1566)	(*n* 4977)		(*n* 399)	(*n* 1192)	(*n* 5275)	

Significant results are indicated in bold font.

A statistically significant gradient in higher risk of both transient and persistent overeating over categories of financial difficulty was seen in age-adjusted and multivariable analyses (Table 4). Interestingly, our multivariable analyses also indicated that underweight in childhood was associated with higher risk of overeating in early adulthood, while prevalence of binge eating was higher among those with a history of childhood overweight. We also saw a higher risk of overeating among binge drinkers (Table 4).

**Table 4:** Relative risk ratios* (RRR) of transient and persistent overeating episodes and binge eating, by sociodemographic and behavioural characteristics, among 6866 Australian women followed longitudinally from 18–23 years of age, Australian Longitudinal Study on Women’s Health

	Overeating								Binge eating							
	Minimally adjusted				Multivariable analysis				Minimally adjusted				Multivariable analysis			
	Persistent		Transient		Persistent		Transient		Persistent		Transient		Persistent		Transient	
	RRR	95 % CI	RRR	95 % CI	RRR	95 % CI	RRR	95 % CI	RRR	95 % CI	RRR	95 % CI	RRR	95 % CI	RRR	95 % CI
Parental education	*P*=0·763								***P*** **<0**·**001**				***P*** =**0**·**028**			
Up to 12 years	1		1		–		–		**1**		**1**		**1**		**1**	
Trade/certificate/diploma	1·07	0·79, 1·44	0·98	0·85, 1·14					**0**·**69**	**0**·**52**, **0**·**91**	**0**·**84**	**0**·**71**, **1**·**00**	**0**·**72**	**0**·**54**, **0**·**96**	**0**·**88**	**0**·**74**, **1**·**04**
Degree/higher degree	1·10	0·82, 1·50	1·02	0·87, 1·18					**0**·**93**	**0**·**71**, **1**·**22**	**0**·**95**	**0**·**80**, **1**·**13**	**0**·**93**	**0**·**70**, **1**·**23**	**0**·**99**	**0**·**83**, **1**·**18**
Not known	0·96	0·68, 1·35	0·88	0·74, 1·05					**1**·**24**	**0**·**94**, **1**·**65**	**1**·**25**	**1**·**04**, **1**·**50**	**1**·**15**	**0**·**85**, **1**·**54**	**1**·**15**	**0**·**95**, **1**·**38**
Weight in childhood	***P*** **<0**·**001**				***P*** **<0**·**001**				***P*** **<0**·**001**				***P*** **<0**·**001**			
Underweight	**1**·**47**	**1**·**14**, **1**·**89**	**1**·**23**	**1**·**07**, **1**·**41**	**1**·**45**	**1**·**12**, **1**·**88**	**1**·**22**	**1**·**07**, **1**·**40**	**0**·**84**	**0**·**63**, **1**·**13**	**0**·**96**	**0**·**81**, **1**·**12**	**0**·**99**	**0**·**74**, **1**·**34**	**1**·**04**	**0**·**88**, **1**·**23**
Average	**1**		**1**		**1**		**1**		**1**		**1**		**1**		**1**	
Overweight	**0**·**75**	**0**·**55**, **1**·**03**	**0**·**89**	**0**·**77**, **1**·**03**	**0**·**73**	**0**·**53**, **0**·**99**	**0**·**88**	**0**·**76**, **1**·**01**	**2**·**66**	**2**·**12**, **3**·**34**	**1**·**77**	**1**·**52**, **2**·**05**	**2**·**10**	**1**·**66**, **2**·**66**	**1**·**53**	**1**·**31**, **1**·**78**
Residence	*P*=0·176								*P*=0·051				***P*** =**0**·**012**			
Major cities	1		1		–		–		1		1		**1**		**1**	
Inner regional	1·04	0·81, 1·34	1·08	0·95, 1·23					0·76	0·60, 0·97	0·90	0·78, 1·04	**0**·**72**	**0**·**56**, **0**·**92**	**0**·**88**	**0**·**76**, **1**·**03**
Outer regional	0·69	0·47, 1·02	1·16	0·98, 1·37					0·64	0·46, 0·91	1·01	0·84, 1·22	**0**·**57**	**0**·**40**, **0**·**82**	**0**·**98**	**0**·**81**, **1**·**20**
Remote	0·81	0·39, 1·69	0·99	0·70, 1·39					1·15	0·67, 1·99	0·95	0·64, 1·39	**1**·**04**	**0**·**59**, **1**·**82**	**0**·**91**	**0**·**61**, **1**·**35**
Language	*P*=0·298								***P*** =**0**·**007**				***P*** =**0**·**032**			
English, Australian	1		1		–		–		**1**		**1**		**1**		**1**	
English, other	1·24	0·78, 1·96	0·83	0·63, 1·09					**1**·**37**	**0**·**90**, **2**·**07**	**1**·**16**	**0**·**88**, **1**·**54**	**1**·**35**	**0**·**88**, **2**·**08**	**1**·**18**	**0**·**89**, **1**·**57**
European	0·64	0·32, 1·25	1·03	0·78, 1·35					**1**·**63**	**1**·**05**, **2**·**52**	**1**·**66**	**1**·**26**, **2**·**19**	**1**·**46**	**0**·**92**, **2**·**32**	**1**·**60**	**1**·**20**, **2**·**14**
Asian or other	1·22	0·65, 2·28	1·25	0·90, 1·72					**0**·**78**	**0**·**40**, **1**·**55**	**1**·**07**	**0**·**74**, **1**·**54**	**0**·**80**	**0**·**39**, **1**·**60**	**1**·**14**	**0**·**78**, **1**·**66**
Education	*P*=0·823								***P*** =**0**·**006**				***P*** =**0**·**036**			
Up to 12 years	1		1		–		**–**		**1**		**1**		**1**		**1**	
Trade/certificate/diploma	1·13	0·83, 1·54	1·02	0·87, 1·20					**0**·**66**	**0**·**48**, **0**·**90**	**0**·**88**	**0**·**74**, **1**·**05**	**0**·**65**	**0**·**47**, **0**·**89**	**0**·**87**	**0**·**73**, **1**·**05**
Degree or higher degree	1·22	0·85, 1·75	1·00	0·83, 1·20					**0**·**74**	**0**·**53**, **1**·**04**	**0**·**76**	**0**·**61**, **0**·**94**	**0**·**86**	**0**·**61**, **1**·**22**	**0**·**85**	**0**·**68**, **1**·**06**
Employment	*P*=0·745								*P*=0·359							
Paid work	1		1		–		–		1		1		–		–	
Unemployed	0·79	0·46, 1·36	0·89	0·68, 1·15					1·13	0·73, 1·77	0·98	0·74, 1·30				
Other	0·93	0·73, 1·18	0·95	0·84, 1·07					1·00	0·80, 1·25	0·87	0·76, 1·00				
Financial difficulty	***P*** =**0**·**013**				***P*** =**0**·**010**				***P*** **<0**·**001**				***P*** **<0**·**001**			
Not too bad/easy	**1**		**1**		**1**		**1**		**1**		**1**		**1**		**1**	
Difficult sometimes	**1**·**17**	**0**·**90**, **1**·**51**	**1**·**10**	**0**·**97**, **1**·**25**	**1**·**17**	**0**·**91**, **1**·**52**	**1**·**10**	**0**·**97**, **1**·**25**	**1**·**46**	**1**·**16**, **1**·**83**	**1**·**38**	**1**·**19**, **1**·**59**	**1**·**34**	**1**·**06**, **1**·**69**	**1**·**30**	**1**·**13**, **1**·**51**
Difficult always	**1**·**54**	**1**·**14**, **2**·**08**	**1**·**22**	**1**·**04**, **1**·**43**	**1**·**56**	**1**·**15**, **2**·**11**	**1**·**23**	**1**·**05**, **1**·**44**	**1**·**70**	**1**·**29**, **2**·**24**	**1**·**68**	**1**·**41**, **1**·**99**	**1**·**33**	**0**·**99**, **1**·**77**	**1**·**47**	**1**·**23**, **1**·**75**
Living arrangements	*P*=0·150								*P*=0·358							
Parents/relatives	1		1		–		–		1		1		–		–	
Partner and/or children	0·96	0·71, 1·30	1·06	0·92, 1·22					0·99	0·76, 1·30	1·13	0·96, 1·32				
Non-family	1·41	1·06, 1·88	1·14	0·98, 1·33					1·29	0·99, 1·68	1·01	0·85, 1·21				
Alone	1·28	0·81, 2·04	1·16	0·91, 1·47					1·10	0·71, 1·69	0·97	0·74, 1·29				
Parity	*P*=0·719								*P*=0·160							
No children	1		1		–		–		1		1		–		–	
One child	1·15	0·67, 1·98	1·19	0·92, 1·56					1·08	0·66, 1·78	1·30	0·97, 1·74				
Two or more children	0·76	0·27, 2·08	0·98	0·62, 1·52					0·68	0·27, 1·68	0·63	0·35, 1·12				
BMI	*P*=0·050								***P*** **<0**·**001**				***P*** **<0**·**001**			
Underweight	1		1		–		**–**		**1**		**1**		**1**		**1**	
Normal	0·89	0·61, 1·29	0·99	0·81, 1·21					**1**·**78**	**1**·**09**, **2**·**90**	**1**·**62**	**1**·**25**, **2**·**10**	**1**·**59**	**0**·**96**, **2**·**61**	**1**·**57**	**1**·**20**, **2**·**05**
Overweight/obese	0·61	0·39, 0·95	0·88	0·70, 1·10					**4**·**93**	**3**·**00**, **8**·**12**	**2**·**73**	**2**·**07**, **3**·**61**	**3**·**72**	**2**·**21**, **6**·**25**	**2**·**34**	**1**·**75**, **3**·**13**
Smoking	*P*=0·091								***P*** **<0**·**001**				***P*** **<0**·**001**			
Non smoker	1		1		–		**–**		**1**		**1**		**1**		**1**	
Ex-smoker	1·38	1·01, 1·89	1·05	0·88, 1·24					**1**·**76**	**1**·**33**, **2**·**34**	**1**·**41**	**1**·**18**, **1**·**69**	**1**·**61**	**1**·**20**, **2**·**16**	**1**·**32**	**1**·**10**, **1**·**60**
Smoker	1·27	0·98, 1·64	1·12	0·99, 1·28					**1**·**72**	**1**·**37**, **2**·**16**	**1**·**48**	**1**·**29**, **1**·**71**	**1**·**33**	**1**·**04**, **1**·**71**	**1**·**27**	**1**·**09**, **1**·**49**
Alcohol binge drinking	***P*** =**0**·**026**				***P*** =**0**·**041**				***P*** **<0**·**001**				***P*** **<0**·**001**			
No	**1**		**1**		**1**		**1**		**1**		**1**		**1**		**1**	
Yes	**1**·**35**	**1**·**02**, **1**·**78**	**1**·**16**	**1**·**00**, **1**·**34**	**1**·**33**	**1**·**00**, **1**·**75**	**1**·**15**	**0**·**99**, **1**·**33**	**1**·**75**	**1**·**38**, **2**·**23**	**1**·**34**	**1**·**15**, **1**·**57**	**1**·**69**	**1**·**31**, **2**·**19**	**1**·**27**	**1**·**07**, **1**·**50**

Minimally adjusted: adjusted for age at start of follow-up. Multivariable analysis: adjusted for age at start of follow-up and mutually adjusted for other variables shown in the column. Significant results are indicated in bold font.

* No overeating and no binge eating are baseline categories.

Age-adjusted and multivariable analyses of persistent and transient binge eating (Table 4) showed a possible protective effect of average weight or underweight in childhood, residence in regional Australia, higher education and absence of financial difficulties. Additionally, it was observed that risk of persistent binge eating was lower in daughters of parents with a trade qualification while European-language speakers were at statistically significantly higher risk of transient binge eating. Prevalence of binge eating increased dramatically over categories of BMI measured in the first survey and was higher in current and former smokers and binge drinkers.

## Discussion

In a population-based sample of young Australian women, a third reported overeating or binge eating, 16 % reported binge eating, 10 % reported compensatory behaviours and almost 4 % reported symptoms consistent with BN. Among women followed over 4 years, there were 5 % of women with persistent overeating and 6 % of women with persistent binge eating. Our analyses indicated possible protective effects on binge eating of average weight in childhood, residence in regional areas, higher education and absence of financial difficulty. We also noted strong clustering of persistent binge eating with higher BMI in young adulthood, smoking (current or past) and binge drinking.

While the clustering of binge eating with smoking and excessive alcohol consumption in our study is consistent with earlier reports and reviews on an overlap between ED and substance use disorders^(^ ^21^ ^,^ ^22^ ^)^ and a generally stronger association of substance use with BN compared with AN^(^ ^22^ ^)^, larger epidemiological studies demonstrating these associations in a representative population sample are lacking and our study makes an important contribution by highlighting this rather prevalent co-morbidity pattern. Interestingly, in a recent twin study, a part of the co-morbidity pattern between BN and substance use disorders was shown to be attributable to familial factors^(^ ^23^ ^)^ which may include shared genetic predisposition.

Another body of research has explored personality traits, and food-related impulsivity in particular, as one of the potential mechanisms leading to ED^(^ ^24^ ^,^ ^25^ ^)^. Personality traits have also been extensively studied in relation to substance use disorders^(^ ^26^ ^,^ ^27^ ^)^; however, to what extent a variation in personality traits could contribute to the reported co-morbidity patterns between binge eating and binge drinking or how personality characteristics could be used in prevention or diagnosis of ED^(^ ^28^ ^)^ remains to be established.

While binge eating episodes have been shown to be associated with higher BMI in smaller clinical samples^(^ ^29^ ^)^, our results are intriguing in demonstrating not only associations of binge eating with overweight during childhood and adulthood but also a potentially increased risk of overeating in women reporting underweight in childhood. This points towards the importance of a healthy weight trajectory during early life, although the exact underlying mechanisms and direction of any causal effects still need to be established. Large studies with longitudinal data on weight trajectories may be able to address this.

Our results are also consistent with earlier research that demonstrated higher risk of ED in more urbanised areas^(^ ^30^ ^)^. We speculate that the social patterning of binge eating with financial self-perceived stress being the strongest risk factor, and a lower risk of symptoms of disordered eating observed in women with higher education and those living with their family, may be another demonstration of the role of the affected women’s past or current impulsive behaviour in disease aetiology, but could also indicate the causal effects of environmental exposures and a role of psychosocial mechanisms in causing or exacerbating the symptoms of ED. It is of interest to note that the social gradients in more serious symptoms like binge eating or symptoms consistent with BN appeared to be more pronounced than for episodes of overeating, with some additional inconsistencies in the social patterning of these different outcomes such as an unexpected higher prevalence of overeating among women in paid work.

While most published research on ED comes from North American and European populations, it is accepted that ED occur in all cultures but the incidence of anorexia is higher among individuals who have been exposed to Western culture and values and those who live in relative affluence^(^ ^31^ ^)^. Interestingly, Keel and Klump^(^ ^32^ ^)^ argue that BN is a culture-bound syndrome while AN is not. They predict that heritability estimates for bulimia may show greater variability cross-culturally than heritability estimates for anorexia and that genetic bases of these disorders may be associated with differential pathoplasticity. Limited by the design of our study, we find it difficult to speculate about reasons behind the apparently increased risk of binge eating and symptoms consistent with BN in young women from families recently emigrated from Europe in our study.

Unfortunately, the definitions of symptoms of disordered eating and methods of measurement in our study are not fully comparable with earlier investigations among the general population in South Australia by Hay *et al*.^(^ ^12^ ^,^ ^13^ ^)^. The higher prevalence of binge eating as well as compensatory behaviours reported in our study may thus be attributed not only to younger age of our study subjects but also to our focus on behaviours experienced any time during the last month preceding the respective questionnaire surveys.

Our study is a large, population-based study based on a representative sample of young Australian women. Using the self-completed questionnaires, we were able to evaluate the burden of symptoms of disordered eating, including undiagnosed conditions and their milder, pre-clinical forms, as well as the stability and persistence of some symptoms. However, the ALSWH was not designed primarily to investigate ED in a longitudinal perspective and the change of questions included in the respective surveys limits our possibilities for more detailed investigations of the dynamics of the symptoms over time or exploration of the natural history of the disease in this population. Weight in childhood and adulthood and height of the study subjects are also self- reported. This may lead to misclassification and the degree of misclassification, especially for the retrospectively reported weight in childhood, may be greater among those with prevalent ED.

The formal diagnostic criteria for ED have been subject to repeated reviews and changes over the last two decades, reflecting criticism of their failure to classify most clinical presentations, issues in capturing continuities between child, adolescent and adult manifestations, requirements of frequent changes of diagnosis to accommodate the natural course of these disorders as well as disparities among classifications used in clinical compared with research settings^(^ ^31^ ^)^. It has been recently proposed that BN should be extended to include subjective binge eating and that binge eating disorder should be included as a specific diagnostic category defined by subjective or objective binge eating in the absence of regular compensatory behaviour^(^ ^31^ ^)^. The definition of outcome variables in our own analysis is dependent on the specific questions included in the original questionnaire and not entirely consistent with the current formal diagnostic criteria for clinical practice^(^ ^33^ ^)^. However, in the context of the ongoing discussion about distinct aetiological categories, we believe that our analyses of prevalence of overeating episodes and our somewhat relaxed categories of symptoms of ED are justified and well suited to capture even less severe forms of disordered eating with effects on quality of life in a substantial part of the population of young women.

We acknowledge the relatively low response rate of 42 % in the first survey but feel confident that the sample was still well representative of the Australian female population as shown by comparisons with the 1999 and 2001 Australian Census data^(^ ^14^ ^)^. It will be very informative to compare the presented prevalence data with more recent figures from the Australian population and this should be possible once data from the surveys of the new ALSWH 1989–95 cohort become available.

While the completeness of data on the sociodemographic characteristics as well as the questions used to construct our outcome variables was very good, there was 4–14 % of missing data on BMI and smoking. All analyses were restricted to the sample of women with no missing data on any of the variables used in the respective models (complete case analysis). When comparing the sociodemographic characteristics and prevalence of different types of ED between the complete cases and the group excluded from the final analyses, we did not find differences that would indicate major bias in our results. Therefore we believe that our results on patterning of symptoms of disordered eating in young women are valid and may be also relevant for concurrent and recent European female populations as well as other high-income countries.

In our view, the main contribution of our study is in highlighting the large burden of both transient and persistent milder and undiagnosed forms of overeating and disordered eating in the population of young Australian women, in documenting the increased risk of symptoms of disordered eating among socially disadvantaged women (women with lower education, women experiencing financial difficulty and those not living with their families) and in drawing attention to the clustering of unhealthy behaviours. This may help in identifying groups of young women who are in need of early diagnosis and secondary prevention. We also hope the results will attract attention and stimulate research into the long-term health outcomes and consequences of these common symptoms.

## Conclusion

We conclude that overeating, binge eating, compensatory behaviours and symptoms consistent with BN are very common among young Australian women and tend to cluster with smoking and binge drinking. Perceived financial stress and lack of social support appear to increase the risk of binge eating and BN among young Australian women. It is not known whether women of European origin and those with a history of childhood overweight/obesity carry higher risk of bulimia because of genetic or cultural reasons.
